# Influence of Cu on modifying the beta phase and enhancing the mechanical properties of recycled Al-Si-Fe cast alloys

**DOI:** 10.1038/s41598-017-05937-2

**Published:** 2017-07-18

**Authors:** C. B. Basak, N. Hari Babu

**Affiliations:** BCAST, Brunel University London, Kingston Lane, Uxbridge, Middlesex UB8 3PH UK

## Abstract

High iron impurity affects the castability and the tensile properties of the recycled Al-Si alloys due to the presence of the Fe containing intermetallic β-Al_9_Fe_2_Si_2_ phase. To date only Mn addition is known to transform the β-Al_9_Fe_2_Si_2_ phase in the Al-Si-Fe system. However, for the first time, as reported here, it is shown that β-phase transforms to the ω-Al_7_Cu_2_Fe phase in the presence of Cu, after solutionization at 793 K. The ω-phase decomposes below 673 K resulting into the formation of θ-Al_2_Cu phase. However, the present thermodynamic description of the Al-Si-Fe-Cu system needs finer tuning to accurately predict the stability of the ω-phase in these alloys. In the present study, an attempt was made to enhance the strength of Al-6wt%Si-2wt%Fe model recycled cast alloy with different amount of Cu addition. Microstructural and XRD analysis were carried out in detail to show the influence of Cu and the stability range of the ω-phase. Tensile properties and micro-hardness values are also reported for both as-cast and solutionized alloys with different amount of Cu without and with ageing treatment at 473 K. The increase in strength due to addition of Cu, in Fe-rich Al-Si alloys is promising from the alloy recyclability point of view.

## Introduction

The energy requirement of primary aluminium production from bauxite ore is about 186 MJ per kg of metallic aluminium. However, this expenditure could be reduced to 10–20 MJ/kg by recycling the discarded aluminium products or scraps^1, 2^. The recycled aluminium accumulates different metallic impurities due to diversified sources of scraps and involvement of processing equipment, which in turn downgrade the quality of recycled aluminium^3–6^. Despite employing various physical separation techniques^7, 8^, it is inevitable to have residual iron impurity in the scraps, which affects the properties of the recycled aluminium alloys because of two main reasons. Firstly, higher iron content usually associated with shrinkage porosities in the casting^9–13^ as well as lower flowability of liquid metal^14^. Secondly, it reduces the ductility due to the formation of the intermetallic monoclinic β-Al_9_Fe_2_Si_2_ phase, which acts as the stress raiser or crack initiator^15–19^. It has been recently pointed out that properties of high Fe containing recycled Al-Si casting alloys can be improved by changing the morphology of the β-Al_9_Fe_2_Si_2_ phase^20, 21^. However, changing morphology by partial spheroidization of beta phase also makes it completely incoherent with the matrix causing reduction in the strength^22^. The idea behind the present work was to see if the well-known strengthening effect of Cu addition^23^ compensates the effect of softening effect arises due to the high temperature spheroidizing treatment. Most of the available literatures report the studies on Al-Si-Cu alloys having Fe content below 1 wt%^12, 17, 24^. However, in recycled Al-Si alloys iron impurity could be as high as 1.5 wt% or more, therefore, 2 wt% Fe was chosen as the upper ceiling for the level of iron impurity^2, 3^. In the present study, the Cu content was varied from 0 to 6 wt% with an interval of 2 wt% keeping Si content fixed at 6 wt%. A systematic investigation was carried out to study the evolution of phases and resulting microstructure during the heat treatments along with the thermodynamic calculation within the CALculation of PHAse Diagram (CALPHAD) framework. Eventually, microhardness and tensile testing was carried out and correlated with the microstructure to get a full picture about the influence of Cu in Al-Si-Fe alloy system.

## Experimental procedure

Four alloys were selected for the present study, as mentioned earlier, Al-6wt% Si-2wt% Fe with varying Cu content. For the sake of brevity, these alloys would be referred as 0Cu, 2Cu, 4Cu and 6Cu, respectively, in the rest of the article. Al ingots (from Norton Aluminium Ltd.), Al-50wt% Si, Al-40wt% Cu and Al-45wt% Fe master alloys (all from KBM Affilips) having commercial purity level were used for the feed stock. A resistance furnace and boron nitride coated clay bonded graphite crucible was used for preparing the alloy. First Al ingot was melt and then Al-Si, Al-Fe and Al-Cu master alloys were added sequentially with stirring. A melt of about 1.2 kg was prepared at 1073 K with intermittent stirring at an interval of ten minutes and a total holding time of 2 hr. at that temperature to ensure chemical homogeneity. Finally, the melt was conditioned at a temperature of 1023 K and commercially available C_2_Cl_6_ degassing tablet was used. The top oxide layer of the melt was skimmed off before pouring into a boron nitride coated mild steel die (168 mm × 100 mm × 40 mm) preheated at 523 K. An average cooling rate of about 4–5 K/s is expected based on previous experiments and existing literature^25^. Chemical macro-analysis was carried out using glow discharge optical emission spectroscopy (GD-OES) and by micro-analysis using energy dispersive spectroscopy (EDS) attached to a field emission scanning electron microscope (FESEM). Both analysis report the average value from five random locations of the cast slab and reasonable agreement exists between these results; as presented in Supplementary Table S1.

For microstructural analysis, standard metallographic practice was adopted where final polishing was carried out using 0.25 μm colloidal silica. Aqueous solution of 10 wt% NaOH was used for chemical etching, whenever required. The FESEM was equipped with both secondary electron (SE) and back scattered electron (BSE) detectors. The attached energy dispersive spectroscope (EDS) was used for phase specific chemical analysis with 25 kV acceleration voltage and 30 μm objective aperture; for elemental mapping the aperture was increased to 60 μm. Phase specific EDS analysis were carried out on 10 random locations for each phase (with a dwelling time of 120 s for each measurement) and finally average composition along with standard deviation was calculated. Electron backscattered diffraction (EBSD) camera fitted into SEM was used to capture high quality Kikuchi patterns from 10 random locations. Further, these Kikuchi patterns were indexed with the Orientation Image Mapping (OIM) software and directly compared to determine crystallographic orientation between two given phases. Image analysis was carried out using ImageJ software^26^. X-ray diffractometer was equipped with solid-state detector and operated at 1.6 kW Cu-*K*_α_ radiation and Rietveld analysis of the XRD traces was carried out to determine the phases and their lattice parameters using GSAS computer program^27^ with EXPGUI interface^28^ and the texture effect was incorporated using spherical harmonic functions^29^. Thermodynamic calculations were performed with CALPHAD methodology using commercially available PandaT software^30^. Vickers microhardness testing was carried out on the 10 locations across the grip of the tensile specimens using 0.5 kg load with a dwell time of 10 s. This ensures better correlation between the micro-hardness and the tensile properties. Tensile testing was carried out in selected specimens with 25 mm gauge length in compliance with ASTM B557-10 standard^31^. Two numbers of tensile testing were carried out for each treatment and the better one has been reported here.

## Results

**Figure 1:**
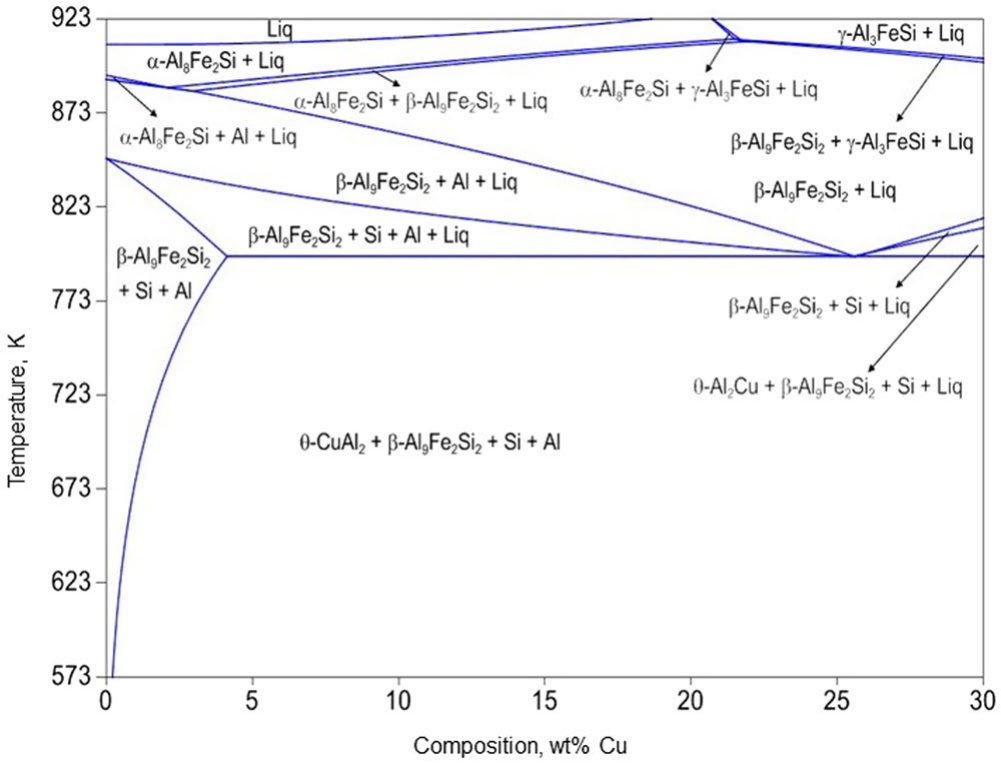
Computed isopleth at a section of 6 wt% Si-2 wt% Fe. Note the absence of ω-Al_7_Fe_2_Cu phase under equilibrium condition upto 30 wt% Cu.

Average chemical composition of the alloys was determined by GD-OES and EDS analysis are presented in Supplementary Table S1. The isopleth at 6wt%Si-2wt%Fe was calculated using CALPHAD methodology and is presented in Fig. 1. Microstructural and XRD results for the as-cast, solutionized and aged conditions are presented below under the corresponding sub-headings.

### As-cast structure

**Figure 2:**
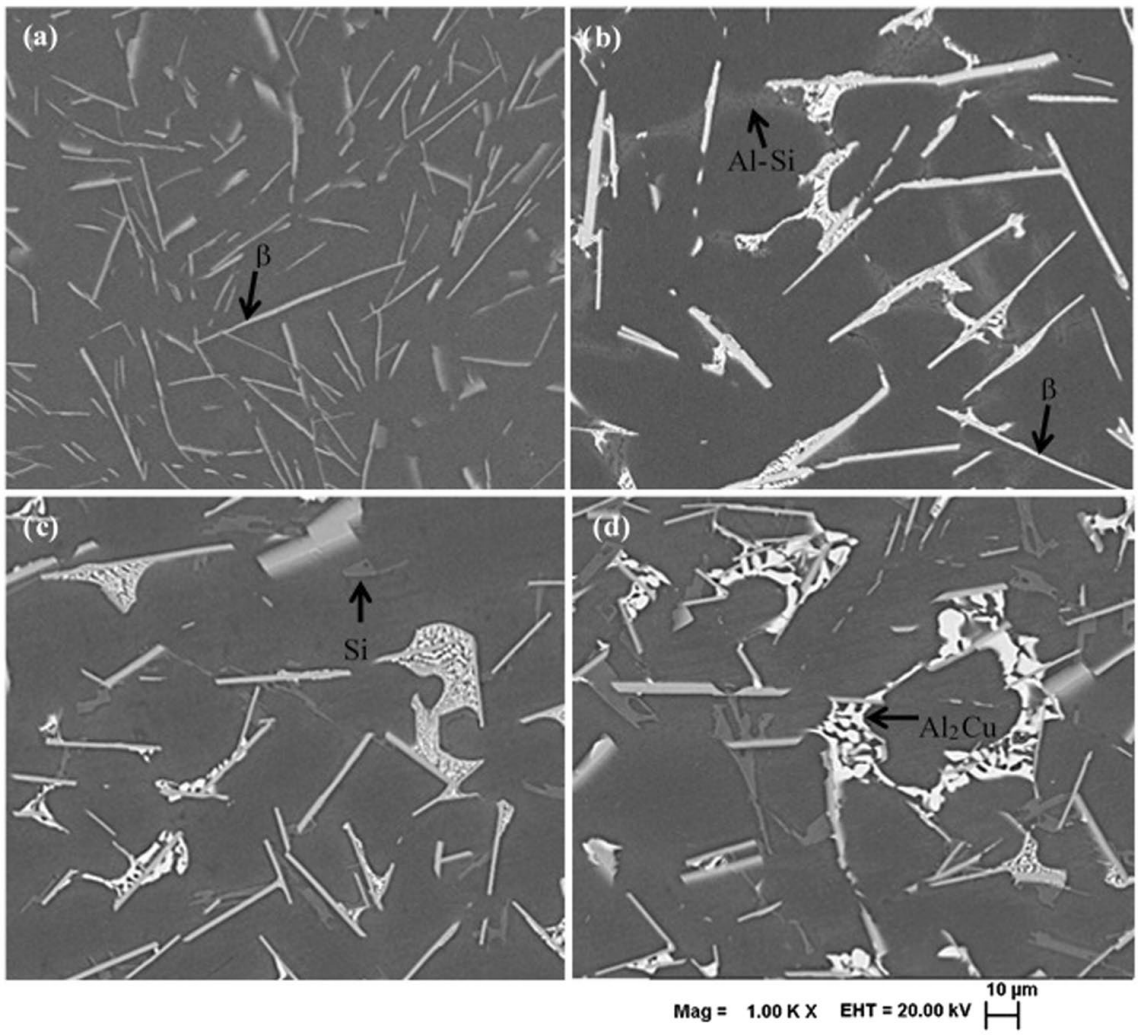
BSE images of as-cast microstructures of (**a**) 0Cu, (**b**) 2Cu, (**c**) 4Cu and (**d**) 6Cu samples showing needle like β-phase. Small amount of fine Al-Si eutectic was observed in 0Cu and 2Cu samples, however, primary Si phase is predominant in 4Cu and 6Cu samples along with θ-Al_2_Cu eutectic phase.

Typical as-cast microstructures of these alloys are presented in Fig. 2(a–d). Average chemical compositions and corresponding standard deviations for each phase, derived from EDS analysis, are presented in Supplementary Table S2. Typical Kikuchi patterns of β-Si and θ-β phase pairs are presented in Supplementary Fig. S1 for the checking of parallelism between different crystallographic planes belonging to two different phases. Whole pattern fitting of the XRD traces using Rietveld analysis for the cast alloys are presented in Supplementary Fig. S2. The calculated lattice parameters and volume fraction of individual phases are presented in Table 1 as obtained from the fitting. Image analysis also carried out from the micrographs of the as-cast sample to determine the area fraction of the Al-matrix (Supplementary Fig. S3); which is found to be 79.7%, 75.2% and 72.5% for 2Cu, 4Cu and 6Cu alloys, respectively (Table 1).

**Table 1:** Lattice parameters (×10 nm) and phase fraction (vol%) of different phases of as-cast (AC) and solutionized (Sol) sample from XRD analysis with R_p_, Rw_p_ and χ^2^ parameters indicating goodness of fitting.

Sample	Al (matrix)			Si		Al_9_Fe_2_Si_2_ (β)					Al_2_Cu (θ)			Al_7_Cu_2_Fe (ω)			R_p_	R_wp_	χ^2^
	*a*	*vol%*	*area%*	*a*	*vol%*	*a*	*b*	*c*	*β*	*vol%*	*a*	*c*	*vol%*	*a*	*c*	*vol%*			
Literature	4.0496	—	—	5.4309	—	20.8130	6.175	6.161	90.42	—	6.0670	4.8700	—	6.3360	14.8700	—	—	—	—
2Cu_AC	4.0444	86.59	79.66	5.4256	5.59	20.8091	6.1813	6.1629	90.45	5.88	6.0595	4.8724	1.94	—	—	—	0.06	0.89	8.28
4Cu_AC	4.0425	83.76	75.24	5.4220	5.65	20.7634	6.1721	6.1532	90.38	6.21	6.0658	4.8824	4.38	—	—	—	0.09	0.16	9.59
6Cu_AC	4.0425	81.18	72.48	5.4300	5.86	20.7843	6.1744	6.1594	90.41	6.10	6.0616	4.8816	6.86	—	—	—	0.05	0.07	7.40
2Cu_Sol	4.0447	89.44	83.41	5.4311	4.69	20.7729	6.1652	6.1632	90.18	5.87	—	—	—	—	—	—	0.09	0.06	7.28
4Cu_Sol	4.0423	88.67	81.39	5.4315	4.78	20.8067	6.1732	6.1622	90.30	4.37	—	—	—	6.3333	14.8001	2.18	0.06	0.08	7.59
6Cu_Sol	4.0405	86.31	78.86	5.4297	4.92	20.8116	6.1741	6.1622	90.30	2.19	—	—	—	6.3219	14.8099	6.58	0.04	0.06	4.06

Solutionized condition implies soaking at 793 K for 684 ks and then water quenched. Area% indicated under Al (matrix) column is the result from the image analysis.

### Solutionized structure

**Figure 3:**
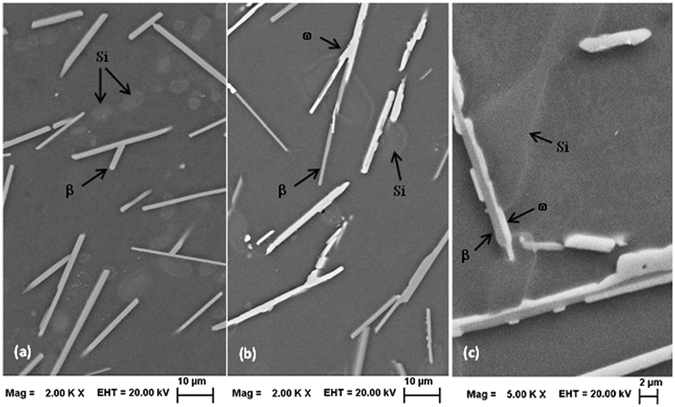
BSE image of the samples solutionized at 793 K for 86.4 ks; (**a**) 2Cu sample showing needle like β-Al_9_Fe_2_Si_2_ phase and Si (arrow) (**b**) 4Cu and (**c**) 6Cu sample showing ω-Al_7_Cu_2_Fe phase (brighter contrast) with remaining β-Al_9_Fe_2_Si_2_ phase (arrows); atomic number contrast is apparent between these two phases. The θ-Al_2_Cu phase was absent in all solutionized samples.

**Figure 4:**
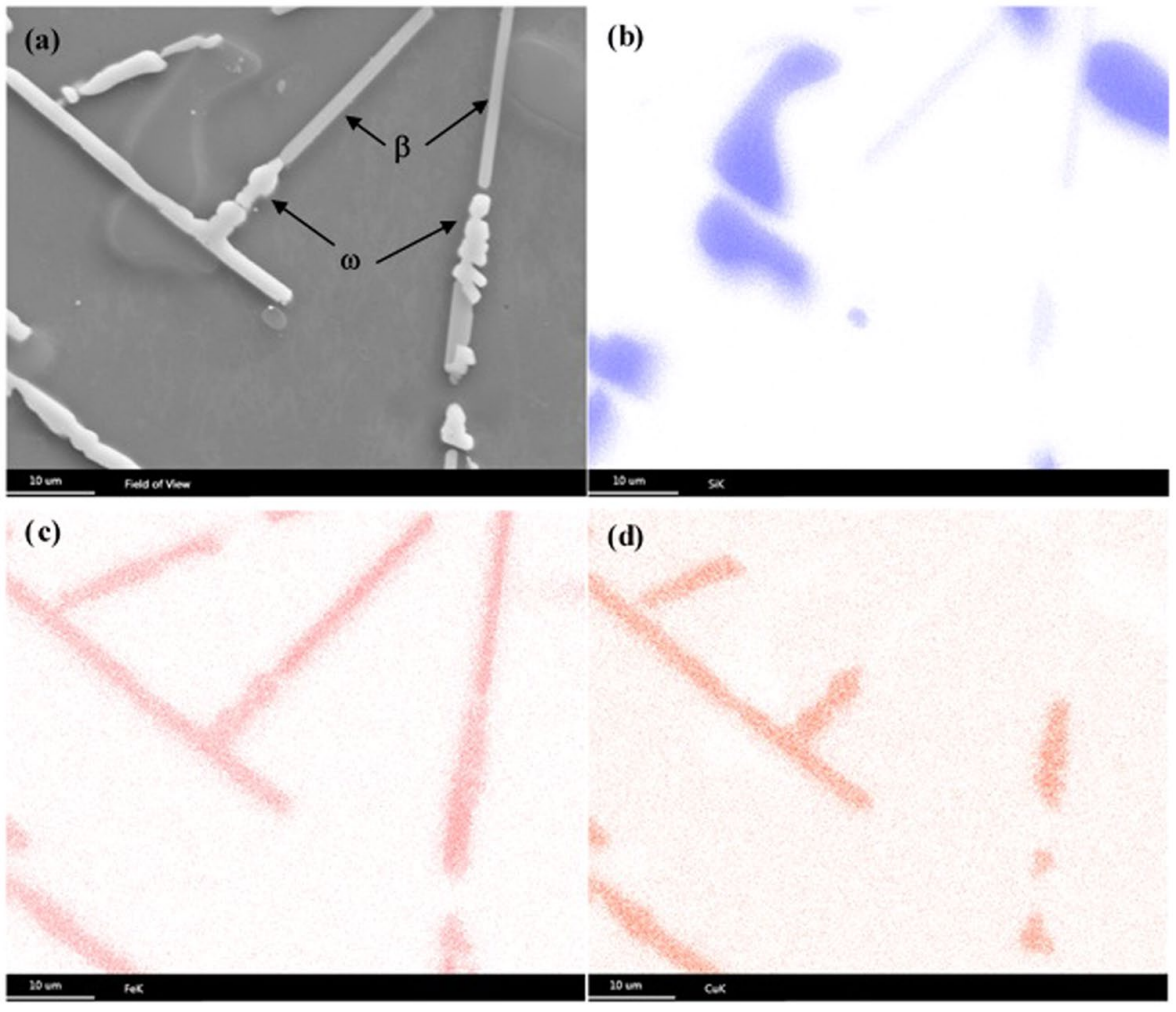
(**a**) SEM micrograph of 6Cu sample solutionized at 793 K for 684 ks and (**b**–**d**) corresponding x-ray elemental mapping (with EDS) using, (**b**) Si-K_α_, (**c**) Fe-K_α_ and (**d**) Cu-K_α_. Si, Al_9_Fe_2_Si_2_ (β) and Al_7_Cu_2_Fe (ω) phases were identified along with Al-matrix but Al_2_Cu (θ) phase was not observed in the microstructure.

Solutionization of 2Cu, 4Cu and 6Cu samples were carried out at 793 K, below the quaternary eutectic temperature (797 K), from 7.2 ks to 684 ks and then quenched in the water. The corresponding XRD traces are presented in Supplementary Fig. S4. Whole pattern fitting for the solutionized samples (793 K for 684 ks) were carried out (Supplementary Fig. S5) to determine the lattice parameters and phase fractions for all the phases as presented in Table 1. Image analysis reveals the area fraction of the Al-matrix is 83.4%, 81.4% and 78.9% for 2Cu, 4Cu and 6Cu alloy samples solutionized for 684 ks (Supplementary Fig. S6). BSE images of solutionized 2Cu sample is presented in Fig. 3a indicating the absence of ω phase. In 4Cu (Fig. 3b) and 6Cu (Fig. 3c) solutionized sample both ω and β phase was observed; ω-phase being the major phase in 6Cu sample. The elemental mapping using EDS was carried out on the solutionized 6Cu sample (793 K for 684 ks), for the direct observation of co-existing β and ω phase as presented in Fig. 4(a–d).

### Aged structure

**Figure 5:**
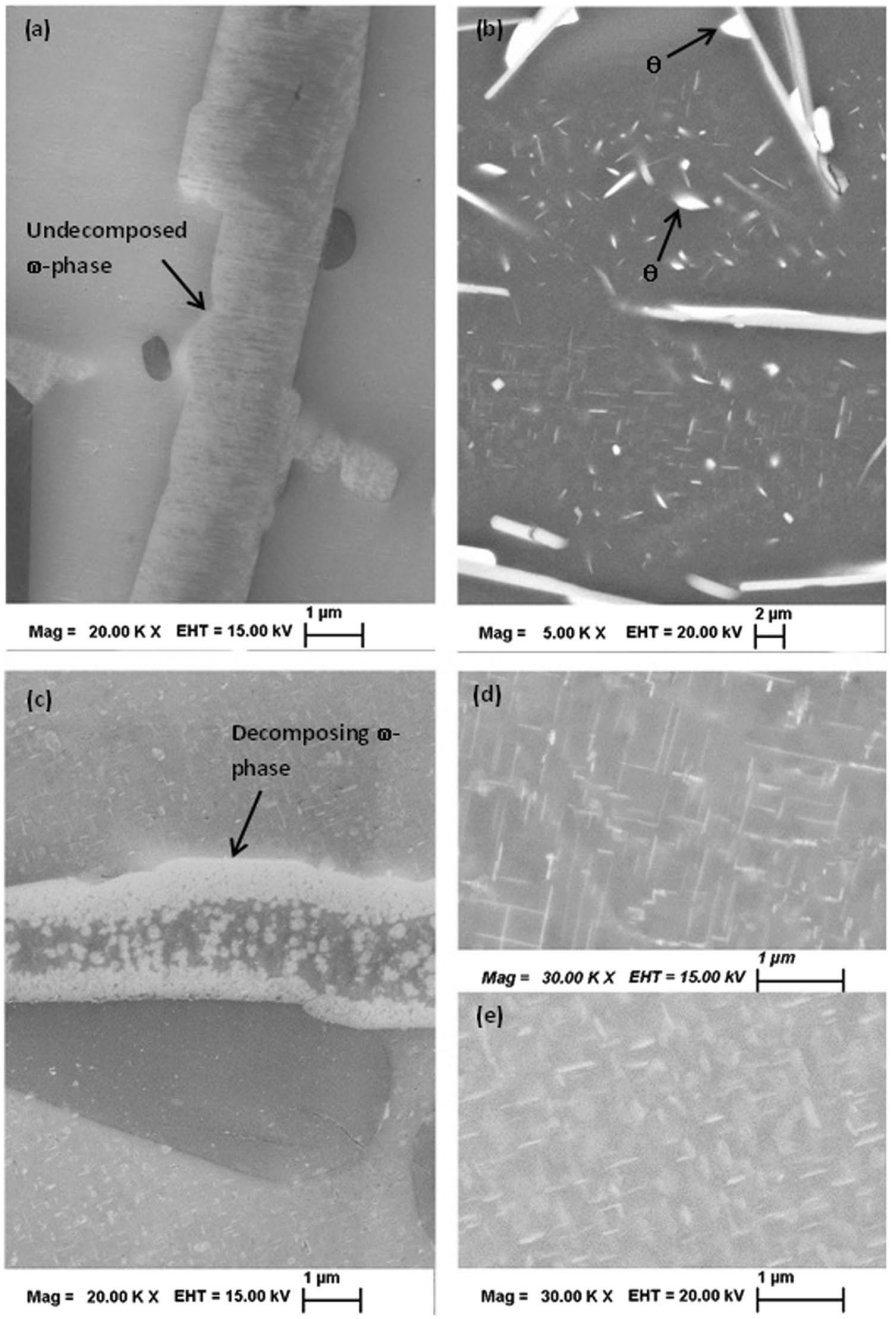
(**a**) In-lens SE image of 6Cu sample aged at 673 K for 432 ks showing undecomposed ω-phase (arrow); (**b**) BSE image of 4Cu sample aged at 573 K for 864 ks showing two types of θ-Al_2_Cu precipitates, in matrix and adjacent to the β-phase (marked by arrows) (**c**) In-lens SE image 6Cu sample aged at 473 K for 260 ks showing decomposing ω-phase (arrow). BSE image of 6Cu sample showing θ^/^precipitates in the matrix aged at 473 K for (**d**) 260 ks and (**e**) 950 ks. Number density of θ^/^precipitates is about 12/μm^2^ and 10/μm^2^ for (**d**) and (**e**), respectively.

The samples of 2Cu, 4Cu and 6Cu alloys were first solutionized at 793 K for 86.4 ks and then aged at 773 K, 673 K, 573 K and 473 K for 260 ks, 432 ks, 864 ks and 950 ks respectively, to achieve expected equilibration. XRD traces of the aged samples are presented in Supplementary Fig. S7 and the phases identified from XRD analysis are listed in Table 2. Constituent phases are unambiguously determined by XRD analysis for 4Cu and 6Cu alloy samples homogenized at 793 K and at 773 K; namely, Al, Si, β and ω; whereas the same for 2Cu samples were Al, Si and β phase. Undecomposed ω-phase in 6Cu sample aged at 673 K for 432 ks is presented in Fig. 5a. Figure 5b shows coarse θ-Al_2_Cu particle precipitated on the existing ω or β-phase and fine θ particles in the matrix in 4Cu sample aged at 573 K for 864 ks. The decomposing ω-phase in 6Cu sample aged at 473 K for 260 ks is presented in Fig. 5c (in comparison to Fig. 5a). After aged at 473 K for 260 ks and 950 ks the 6Cu sample shows metastable θ^/^-phase in the microstructure which coarsens with time; as presented in Fig. 5d and e, respectively.

**Table 2:** Detected phases from XRD analysis and corresponding estimated equilibrium phases at different temperatures; bold faced letter indicate confirmed presence of equilibrium ω-phase in different samples.

Temp (K)	2Cu		4Cu		6Cu	
	XRD	Eqm.	XRD	Eqm.	XRD	Eqm.
793	β, Al, Si	β, Al, Si	ω, β, Al, Si	**ω, β, Al, Si**	ω, β, Al, Si	**ω, β, Al, Si**
773	β, Al, Si	β, Al, Si	ω, β, Al, Si	**ω, β, Al, Si**	ω, β, Al, Si	**ω, β, Al, Si**
673	ω, θ, β, Al, Si	**ω, β, Al, Si**	ω, θ, β, Al, Si	**ω, β, Al, Si**	ω, θ, β, Al, Si	**ω, β, Al, Si**
573	ω, θ, β, Al, Si	θ, β, Al, Si	ω, θ, β, Al, Si	θ, β, Al, Si	ω, θ, θ′, β, Al, Si	θ, β, Al, Si
473	ω, θ′, β, Al, Si	θ, β, Al, Si	ω, θ′, β, Al, Si	θ, β, Al, Si	ω, θ′, β, Al, Si	θ, β, Al, Si

### Mechanical properties

**Figure 6:**
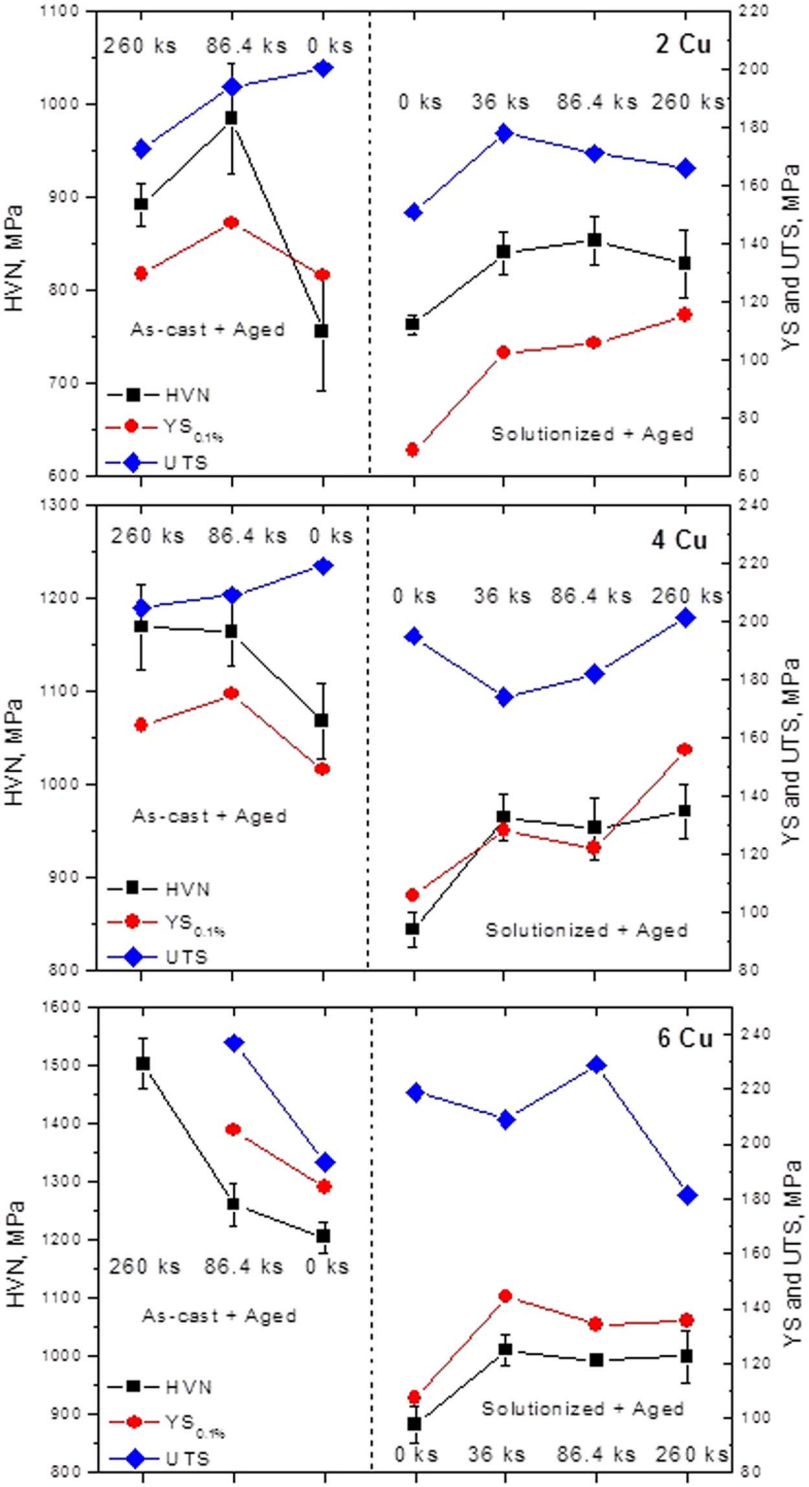
Combined plots for Vickers micro-hardness (HVN), 0.1% offset yield stress (YS) and UTS of 2Cu, 4Cu and 6Cu samples after ageing at 473 K for different durations in as-cast condition (left side of the dashed line) and after solutionization at 793 K for 43.2 ks (right side of the dashed line). Lines connecting the data points are only for the visual guide.

To compare mechanical properties, as-cast and solutionized (at 793 K for 86.4 ks) conditions was taken as starting state and further ageing treatment was carried out with these two initial conditions. Tensile samples were not tested for the presence of any internal porosity or defects; therefore, for all practical purposes the tensile properties would only be better than the values reported here^32^. Experimentally obtained stress-strain curves are presented in the Supplementary Fig. S8. Ultimate tensile strength (UTS), 0.1% offset yield strength (YS) along with Vickers microhardness values are presented in self-explanatory manner in Fig. 6(a–c). For 0Cu alloy the Vickers microhardness values in the as-cast and solutionized condition are virtually identical, i.e. 689.4 and 684.5 MPa respectively. No considerable difference was observed in UTS as well; 156 MPa and 149.7 MPa for as-cast and solutionized condition respectively. However, marked difference in YS was noted; 64.5 MPa for solutionized sample as against 91.8 MPa in as-cast condition. Upon solutionizing treatment 0Cu sample exhibits increase in ductility to about 2.7% from the as-cast condition (1.5%). Solutionization causes partial spheroidization of the β-phase needles and grain growth of the matrix which could be attributed to the increased ductility with lowering YS^21^.

### Data Availability

All metadata pertaining to this work can be accessed via the following link: https://doi.org/10.17633/rd.brunel.5086420.v1

## Discussions

### Cast structures

It is evident from the SEM micrographs (Fig. 2) that the as-cast alloys contain the phases, namely, Al, Si, β-Al_9_Fe_2_Si_2_ and θ-Al_2_Cu, that are in line with the thermodynamically predicted phases (Fig. 1). However, as copper content increases in these alloys, the phase fraction of Al_2_Cu increases and instead of eutectic Al-Si mostly primary Si particle appears in the microstructure. EDS analysis (Supplementary Table S2) reveals an interesting observation regarding the systematic change in the chemical composition of the β-phase (Al_9_Fe_2_Si_2_) in the as-cast samples. As Cu content in the alloy increases, β-phase exhibits higher solubility for Cu and lowering in Si and Fe content. In 6Cu alloy Fe content in the β-phase is just about the half as compared to the 0Cu alloy. However, the lattice parameters of the β-phase does not change significantly due to the enrichment of Cu and simultaneous reduction in Fe and Si.

In earlier study, it was suggested that β-phase provides nucleation site for the eutectic solidification of Si from the liquid^21^. Direct comparison of the Kikuchi patterns for Si- β phase pair (Supplementary Fig. S1) in the as-cast samples reveals that different crystallographic plane of Si (from different location) is parallel to either {933} or $\{\bar{9}11\}$ plane of the adjacent β-phase substrate (usually within 0.2^o^), but no definite orientation relationship was observed between β-phase and Si. On the other hand, no such systematic parallelism of any plane was observed for the β-θ phase pair (Supplementary Fig. S1). Therefore, it can be inferred that β-phase acts merely as heterogeneous nucleation site for the subsequent formation of θ and Si phase without any specific crystallographic orientation relationship.

Systematic discrepancy between area fraction of the matrix obtained from image analysis and the volume fraction obtained from the XRD analysis could be solely attributed to the morphology and distribution of the other phases that indirectly affects the area fraction of the matrix; which is a well-established fact^33^.

### Solutionized structure

It has been seen from the XRD patterns (Supplementary Fig. S4) that ω-Al_7_Cu_2_Fe phase forms just after 7.2 ks of exposure at 793 K in 4Cu and 6Cu sample and the phase content does not change appreciably beyond 86.4 ks of soaking. When compared to the XRD patterns of the as-cast samples, it was observed that Al_2_Cu (θ) phase completely disappeared after the solutionization treatment. This explains the corresponding increase in the area fraction of the matrix, as obtained from the image analysis, as compared to the as-cast structure. Microstructural observations (Fig. 3) and XRD analysis confirms the presence of Al, Si and β-Al_9_Fe_2_Si_2_ phases in all solutionized samples in addition to ω-Al_7_Cu_2_Fe phase in 4Cu and 6Cu samples. The elemental mapping using EDS carried out on the solutionized 6Cu sample (Fig. 4) corroborates the co-existing β and ω phase. SEM micrographs also clearly indicates that most of the existing β-phase needles fully or partially converted into the ω-phase during solutionizing treatment, as presented in Fig. 3(a–c). Microstructural evidences indiactes that the ω-phase forms due to the reaction of β-phase with Cu; where the prime source of Cu is the Al_2_Cu phase which is unstable at the solutionizing temperature. From EDS results (Supplementary Table S2) it could be seen that after solutionizing treatment Al-matrix gets enriched in Cu which is also primarily due to the dissolution of Al_2_Cu phase. However, 2Cu alloy does not show the existence of either θ-phase or ω-phase after the solutionizing treatment at 793 K; because, Cu concentration in the alloy is within the solubility limit of the matrix phase and therefore it is expected that all the Cu atoms would go to matrix rather than forming any intermetallic compound.

It is important to point out that the existing thermodynamic description of Al-Si-Fe-Cu system could not predict the presence of ω-phase at 793 K even upto 30 wt% Cu as evident from the isopleth at 6wt%Si-2wt%Fe (Fig. 1). To eliminate any possible error in the present thermodynamic calculations, a phase stability calculation was carried out at 793 K for Al-6wt% Si-2wt% Fe- 6 wt% Cu alloy; with the latest aluminium database using PandaT and Thermocalc software independently and both predict the same stable phases, namely, Al, Si, β-Al_9_Fe_2_Si_2_ and θ-Al_2_Cu (calculation performed on personal request by Malin Selleby of KTH Royal, Sweden using Thermocalc software and F. Zhang of Computherm LLC, USA with their PandaT software)^34^. Further thermodynamic analysis of Al-Si-Fe-Cu system was carried out at 2 wt% Fe and varying Si content; these isopleths are presented in Supplementary Fig. S9. It could be seen from these isopleths that at low Si content ω-phase appears at higher temperature; in confirmation with the findings of Chen *et al*.^34^. However, ω-phase field ceases to exist in the system above 3 wt% Si. It is equally important to note that with increasing Si content the θ-phase field expands towards higher temperature. Comparison of the experimental observations with these isopleths makes it clear that the stability of θ-phase is overestimated as compared to ω-phase in low Si containing Al-Cu-Fe system. Since, neither θ or ω intermetallic phase contains Si, therefore it is most likely that Fe containing temperature dependent interaction parameters of Gibbs free energy for β and ω phase need further tuning in the present thermodynamic description to establish their correct relative stability.

### Aged structure

4Cu and 6Cu samples solutionized and aged at 773 K exhibit similar phase content, namely, Al, Si, β and ω; whereas the same for 2Cu samples were Al, Si and β phase. However, in the temperature range from 673 K to 473 K, more than 4 phases could be found from the XRD analysis depending on the Cu content and the ageing condition (Table 2); which is in violation of Gibbs phase rule and therefore such phase aggregates cannot be considered as the equilibrium phases. Therefore, further confirmation is required to ascertain the relative stability among the phases. When the solutionized samples are subjected to the low temperature ageing (T ≤ 673 K) two points are worth remembering; first, the matrix is supersaturated with Cu and second, diffusion of Cu in Al is rather sluggish. Calculated diffusivity of Cu shows fourth order decrease in diffusivity from 2.9 × 10^−15^ m^2^/s at 673 K to 2.4 × 10^−19^ m^2^/s at 473 K^35^. These two phenomena promote a local equilibrium, where Cu bearing intermetallic precipitates (θ^/^and/or θ) start forming from the Cu supersaturated Al-matrix through the known sequence of different metastable phases i.e. GP zones → θ^//^ → θ^/^ → θ^36^ or directly without forming any precursor phase depending on the level of Cu-supersaturation^37^. Therefore, mere presence of the θ-phase or ω-phase should not be construed as the thermodynamically stable (i.e. equilibrated) phase.

XRD analysis of 2Cu, 4Cu and 6Cu alloy samples homogenized at 673 K for 432 ks confirms the presence of both θ and ω-phase. However, to ascertain the relative stability between θ and ω phase, another ageing treatment was carried out at the same temperature but for a shorter duration of 260 ks. Comparative XRD analysis after the whole pattern fitting reveals the ω phase fraction actually increases with the ageing time; for example, in 6Cu sample ω-phase content increases from 7.1% to 9.5% when soaking time increases from 260 ks to 432 ks with a decrease in θ content, from 2.3% to 1.8%, at 673 K. Therefore, when aged at 673 K, initially θ-content will increase from 0% (solutionized) to 2.3% (aged for 260 ks) as per the local equilibrium condition and then decrease with time (1.8% after aged for 432 ks). Correspondingly, the phase fraction of ω increases from 6.85% (solutionized) to 7.1% (aged for 260 ks) and 9.5% after aged for 432 ks (Supplementary Fig. S10). Similarly, XRD analysis shows that 2Cu sample contains only θ-phase and both θ and ω phase when aged at 673 K for 260 ks and 432 ks respectively. The fact that at 673 K, ω-phase is stable and not decomposing could also be observed from the micrograph presented in Fig. 5a (compare with decomposing ω-phase in Fig. 5c). From these experimental observations, it could be concluded that at 673 K ω-phase is the thermodynamically stable one.

XRD analysis of 2Cu and 4Cu samples aged at 573 K for 864 ks reveals presence of both θ and ω-phase; in addition to these phase 6Cu sample shows θ^/^phase. Figure 5b shows coarse θ particle precipitated on the existing ω or β-phase and fine θ particles in the matrix in 4Cu sample. Comparison of ω-content in aged 6Cu samples at 673 K and 573 K reveals that the phase fraction reduces from 9.5% (aged at 673 K) to 7.5% (aged at 573 K). All alloy samples homogenized at 473 K for 950 ks exhibit only θ^/^(instead of θ) and ω phase as confirmed by XRD analysis. Just after 260 ks soaking at 473 K the 6Cu sample exhibits decomposing ω-phase as presented in Fig. 5c (in comparison to Fig. 5a). On the other hand, it could be seen that after heating at 473 K for 260 ks and 950 ks the metastable θ^/^-phase remains in the microstructure and only coarsens with time; as evident from Fig. 5d and e, respectively. In the XRD patterns with two prominent peaks corresponding to (101) and (103) planes of tetragonal θ^/^-phase could be observed in 4Cu and 6Cu sample (Supplementary Fig. S7). Evidence of decomposing ω-phase suggests instability of ω-phase and therefore stability of θ-phase at 573 K and 473 K; these findings are summarized in Table 2, under the column named ‘eqm’ denoting expected equilibrium phases. In summary, it could be said with certainty that the thermodynamic instability of the ω-phase in these alloy is below 573 K.

### Mechanical properties and effect of ageing

Overall it was observed that Cu addition decreases ductility but with marked increase in YS and UTS; in general, solutionization improves ductility for 2Cu, 4Cu and 6Cu alloys as compared to their as-cast condition, however, it is never seen to exceed 1.5%. Since, ageing treatment at 473 K promotes θ^/^-phase formation, corresponding increase in hardness, YS and UTS are apparent from Fig. 6. Usually, 86.4 ks of ageing at 473 K exhibit optimum combination of ductility (~1% for solutionized condition and ~0.5% for as-cast condition) along with YS, UTS and hardness. Considering the recyclability of high iron containing Al-Si cast alloy it looks promising to add Cu to improve the YS, UTS and hardness with marginal loss of ductility.

From the EDS results (Supplementary Table S2) it is evident that Cu content is higher in the matrix in solutionized condition than that in as-cast condition. On the other hand, it is known that higher degree of Cu-supersaturation in the matrix causes early onset of the peak hardness upon ageing^38^. This explains why the peak hardness reaches early during ageing for the solutionized 6Cu sample than 4Cu or 2Cu sample. This also explains why solutionized sample attains peak hardness faster that the as-cast sample for the same alloy composition. The micro-inhomogeneity in the as-cast sample is the inherent characteristics attributed to the non-equilibrium process of solidification. 4Cu and 6Cu alloy has larger mushy zone leading to more chemical inhomogeneity during solidification than that for 0Cu or 2Cu alloy; as reflected by the considerable standard deviation in their micro-hardness value (Fig. 6). The difference in micro-hardness values between as-cast and solutionized sample increases with Cu content as shown in Fig. 6; which is attributed to the fact that, after solidification the ageing might have started in the Cu-rich zone in the cast alloys, during the course of natural cooling of the casting. Therefore, upon ageing, further strengthening of the cast alloy is rather obvious; however, they exhibit poor ductility, often less than 0.5% (Supplementary Fig. S7). However, ageing treatment after solutionization restores the ductility with marked improvement in YS and UTS.

From the point of view of recycling Al-Si based cast alloy it is known that the presence of Fe is detrimental to the mechanical properties; as highlighted in the “Introduction”. However, based on the evaluated mechanical properties reported here, it could be said that addition of Cu offers solutionization and ageing treatment which improves YS and UTS considerably as compared to 0Cu as-cast model alloy with similar ductility. However, if compared with the solutionized 0Cu alloy, the improvement in YS and UTS is even better.

## Conclusions

From the foregoing discussions, the following points could be drawn –

i. Cu can be used to thermodynamically destabilize the detrimental β-Al_9_Fe_2_Si_2_ phase in the Al-Si-Fe system and thereby offers an alternative route to the traditional Mn addition.
ii. Cu addition upto 6 wt%, in Al-6wt% Si- 2 wt% Fe model recycled cast alloys, tend to stabilize the ω-Al_7_Cu_2_Fe phase above 573 K and θ-Al_2_Cu phase below that temperature. However, present thermodynamic description of Al-Si-Fe-Cu system needs finer tuning to predict this behaviour accurately.
iii. In the as-cast alloys, definite crystallographic orientation relationship was not observed between the β and Si phase or between the β and θ phase.
iv. The addition of Cu in Al-6wt%Si-2wt%Fe alloy improves the YS, UTS and hardness with marginal loss in ductility. After solutionization and ageing for 86.4 ks the alloy with 6 wt%Cu addition offers same ductility as that of as-cast Al-6wt%Si-2wt%Fe alloy but with almost 50% increase in yield strength.

## Electronic supplementary material

Supplementary

## References

[1] Green, J. A. S. (ed), Aluminum recycling and processing for energy conservation and sustainability, ASM International, USA 15 (2007).

[2] Schmitz, C. (ed), Handbook of aluminium recycling, Vulkan Verlag, Germany 25 (2006).

[3] Das SK, Green JAS, Kaufman JG, Emadi D, Mahfoud M (2010). Aluminium recycling – An integrated industrial approach. JOM.

[4] Galloway, T. J. (ed), TMS, USA, *Light metals* 911 (2006).

[5] Gesing A (2004). Assuring the continued recycling of light metals in end-of-life vehicles: A global perspective. JOM.

[6] Kamavaram V, Mantha D, Reddy RG (2003). Electrorefining of aluminium alloy in ionic liquids at low temperatures. J. Min. Metall..

[7] Gaustad G, Olivetti E, Kirchain R (2012). Improving aluminum recycling: A survey of sorting and impurity removal technologies. Resour. Conserv. Recycl..

[8] Spencer DB (2005). The high-speed identification and sorting of nonferrous scrap. JOM.

[9] Taylor JA (2012). Iron-containing intermetallic phases in Al-Si based casting alloys. Proc. Mat. Sci..

[10] Iwahori H, Takamiya H, Yonekura K, Yamamoto Y, Nakamura M (1988). Influence of iron and manganese on feedability of AC2B aluminum alloy. Casting.

[11] Roy N, Samuel AM, Samuel FH (1996). Porosity formation in Al-9 wt% Si-3 wt% Cu alloy systems: Metallographic observations. Metall. Mater. Trans. A.

[12] Ma Z, Samuel AM, Samuel FH, Doty HW, Valtierra S (2008). A study of tensile properties in Al–Si–Cu and Al–Si–Mg alloys: Effect of β-iron intermetallics and porosity. Mater. Sci. Eng. A..

[13] Taylor J, Schaffer G, StJohn D (1999). The role of iron in the formation of porosity in Al-Si-Cu-based casting alloys: Part I. Initial experimental observations. Metall. Mater. Trans. A.

[14] Taghaddos E, Hejazi MM, Taghiabadi R, Shabestari SG (2009). Effect of iron-intermetallics on the fluidity of 413 aluminum alloy. J. Alloy. Comp..

[15] Shabestari SG (2004). The effect of iron and manganese on the formation of intermetallic compounds in aluminum–silicon alloys. Mat. Sci. Eng. A.

[16] Wang L, Makhlouf M, Apelian D (1995). Aluminium die casting alloys: alloy composition, microstructure, and properties-performance relationships. Int. Mater. Rev..

[17] Yi JZ, Gao YX, Lee PD, Lindley TC (2004). Effect of Fe-content on fatigue crack initiation and propagation in a cast aluminum–silicon alloy (A356–T6). Mater. Sci. Eng. A.

[18] Ji S, Yang W, Gao F, Watson D, Fan Z (2013). Effect of iron on the microstructure and mechanical property of Al–Mg–Si–Mn and Al–Mg–Si diecast alloys. Mater. Sci. Eng. A.

[19] Li Z (2004). Parameters controlling the performance of AA319-type alloys: Part I. Tensile properties. Mater. Sci. Eng. A.

[20] Villeneuve C, Samuel FH (1999). Fragmentation and dissolution of β-Al5FeSi phase during solution heat treatment of Al-13wt%Si-Fe alloys. Int. J. Cast Met. Res..

[21] Basak CB, Haribabu N (2016). Morphological changes and segregation of β-Al_9_Fe_2_Si_2_ phase: A perspective from better recyclability of cast Al-Si alloys. Mater. Des..

[22] Basak, C. B. & Haribabu, N. Improved Recyclability of cast Al-alloys by engineering β-Al9Fe2Si2 phase, ed. Ratvik, A. P. & Spinger International Publishing, *Light Metals* 1139 (2017).

[23] Wilm A (1911). Physikalisch-metallurgische Untersuchungen über magnesiumhaltige Aluminiumlegierungen. Metallurgie.

[24] Puncreobutr C (2014). Influence of Fe-rich intermetallics on solidification defects in Al-Si-Cu alloys. Acta. Mater..

[25] Gorny A, Manickaraj J, Cai Z, Shankar S (2013). Evolution of Fe based intermetallic phases in Al–Si hypoeutectic casting alloys: Influence of the Si and Fe concentrations, and solidification rate. J. Alloy Comp..

[26] Schneider CA, Rasband WS, Eliceiri KW (2012). NIH Image to ImageJ: 25 years of image analysis. Nat. Methods.

[27] Larson AC, Von Dreele RB (2000). General Structure Analysis System (GSAS), Los Alamos National Laboratory Report. LAUR.

[28] Toby BH (2001). EXPGUI: a graphical user interface for GSAS. J. Appl. Cryst..

[29] Bunge HJ (1997). Influence of texture in powder diffraction. Texture Microstruct.

[30] Chen SL (2003). Calculating phase diagram using PandaT and Panengine. JOM.

[31] ASTM B557-10, Standard Test Methods for Tension Testing wrought and cast aluminium and magnesium alloy product, ASTM International, West Conshohocken, PA, www.astm.org (2015).

[32] Mugica GW, Tovio DO, Cuyas JC, González AC (2004). Effect of porosity on the tensile properties of low ductility aluminum alloys. Mat. Res..

[33] Basak CB, Sengupta AK (2004). Development of a FDM based code to determine the 3-D size distribution of homogeneously dispersed spherical second phase from microstructure: a case study on nodular cast iron. Scripta. Mater..

[34] Chen H, Du Y, Xu H, Xiong W (2009). Experimental investigation and thermodynamic modeling of the ternary Al–Cu–Fe system. J. Mater. Res..

[35] Anand MS, Murarka SP, Agarwala RP (1965). Diffusion of copper in nickel and aluminium. J. App. Phys..

[36] Porter, D. A. & Easterling, K. E. Phase transformations in metals and alloys, 2^nd^ ed., Chapman and Hall, 291 (1996).

[37] Sehitoglu H, Foglesong T, Maier HJ (2005). Precipitate effects on the mechanical behavior of aluminum copper alloy: Part 1. experiments. Met. Trans. A.

[38] Silcock JM, Heal TH, Hardy HK (1953). structural ageing characteristics in binary aluminium-copper alloys. J. Inst. Met..

