# Supplementary material for: Influence of Cu on modifying the beta phase and enhancing the mechanical properties of recycled Al-Si-Fe cast alloys
Source: Sci Rep. 2017 Jul 18;7:5779. doi: 10.1038/s41598-017-05937-2 (PMC5516036; doi:10.1038/s41598-017-05937-2)
Supplement: Supplementary file 1 — Supplementary [file 41598_2017_5937_MOESM1_ESM.pdf]

Influence of Cu on modifying the beta phase and enhancing the mechanical properties of recycled Al-Si-Fe cast alloys

C. B. Basak*, N. Hari Babu

BCAST, Brunel University London, Kingston Lane, Uxbridge, Middlesex UB8 3PH, UK

Table S1. Overall chemistry of the alloys from GD-OES (first line) and EDS (sample scanned at 200x) analysis

	Si	Fe	Cu	Ga	Ti	In	Mn	Ni	Ca	Sn	Mg	Zn
0Cu	**6.02**	**1.97**	0.003	0.012	0.011	0.01	0.006	0.006	0.006	0.005	0.004	0.004
	**6.16**	**1.96**	--	--	--	--	--	--	--	--	--	--
2Cu	**6.14**	**1.96**	**2.07**	0.011	0.013	0.01	0.007	0.007	0.005	0.007	0.005	0.009
	**6.20**	**1.96**	**1.91**	--	--	--	--	--	--	--	--	--
4Cu	**5.98**	**1.96**	**4.05**	0.014	0.018	0.02	0.005	0.005	0.008	0.004	0.008	0.002
	**6.33**	**2.10**	**3.87**	--	--	--	--	--	--	--	--	--
6Cu	**6.21**	**1.95**	**6.10**	0.0123	0.014	0.0133	0.006	0.006	0.0063	0.0053	0.0057	0.005
	**6.51**	**1.95**	**5.79**	--	--	--	--	--	--	--	--	--

Table S2. Average composition derived from EDS analysis (from 10 random points), along with standard deviation, of different phases in as-cast and solutionized conditions.

Condition	Phase	Sample	Composition, av. wt% ± SD (av. at%)		
			Si	Fe	Cu
As-cast	Matrix	0Cu	0.66 ± 0.30 (0.63)	0.18 ± 0.16 (0.09)	--
		2Cu	0.58 ± 0.27 (0.85)	0.13 ± 0.08 (0.06)	0.93 ± 0.46 (0.39)
		4Cu	0.52 ± 0.31 (0.79)	0.17 ± 0.12 (0.08)	1.28 ± 1.11 (0.55)
		6Cu	0.62 ± 0.35 (0.60)	0.15 ± 0.10 (0.07)	2.24 ± 1.03 (0.96)
	$Al_9Fe_2Si_2$ (β)	0Cu	13.92 ± 0.08 (15.74)	28.00 ± 0.09 (15.92)	--
		2Cu	12.93 ± 0.19 (14.31)	23.56 ± 0.2 (13.11)	0.88 ± 0.16 (0.43)
		4Cu	11.61 ± 0.50 (12.67)	21.02 ± 0.8 (11.54)	1.15 ± 0.15 (0.55)
		6Cu	9.54 ± 0.21 (10.07)	14.16 ± 0.4 (7.52)	2.28 ± 0.18 (1.06)
	Al_2Cu (θ)	2Cu	0.40 ± 0.09 (0.54)	0.71 ± 0.14 (0.49)	50.54 ± 0.98 (30.42)
		4Cu	0.39 ± 0.13 (0.53)	0.71 ± 0.17 (0.49)	51.12 ± 0.66 (30.92)
		6Cu	0.28 ± 0.12 (0.39)	0.69 ± 0.26 (0.48)	53.64 ± 0.19 (33.12)
Solutionized @ 793 K for 684 ks	Matrix	0Cu	0.92 ± 0.37 (0.88)	0.11 ± 0.10 (0.05)	--
		2Cu	0.98 ± 0.17 (0.96)	0.17 ± 0.08 (0.08)	3.12 ± 0.34 (1.35)
		4Cu	1.01 ± 0.28 (0.90)	0.11 ± 0.08 (0.05)	3.20 ± 0.27 (1.39)
		6Cu	0.95 ± 0.27 (0.50)	0.27 ± 0.12 (0.13)	3.44 ± 0.54 (1.49)
	$Al_9Fe_2Si_2$ (β)	0Cu	13.46 ± 0.10 (15.30)	28.91 ± 0.13 (16.52)	--
		2Cu	12.74 ± 0.23 (13.83)	20.21 ± 0.10 (11.04)	1.02 ± 0.10 (0.49)
		4Cu	11.01 ± 0.24 (11.83)	17.52 ± 0.70 (9.47)	1.92 ± 0.21 (0.91)
	Al_7Cu_2Fe (ω)	4Cu	0.59 ± 0.23 (0.76)	12.74 ± 0.40 (8.20)	31.86 ± 0.89 (18.02)
		6Cu	0.33 ± 0.29 (0.43)	13.54 ± 0.86 (8.86)	33.35 ± 0.55 (19.19)

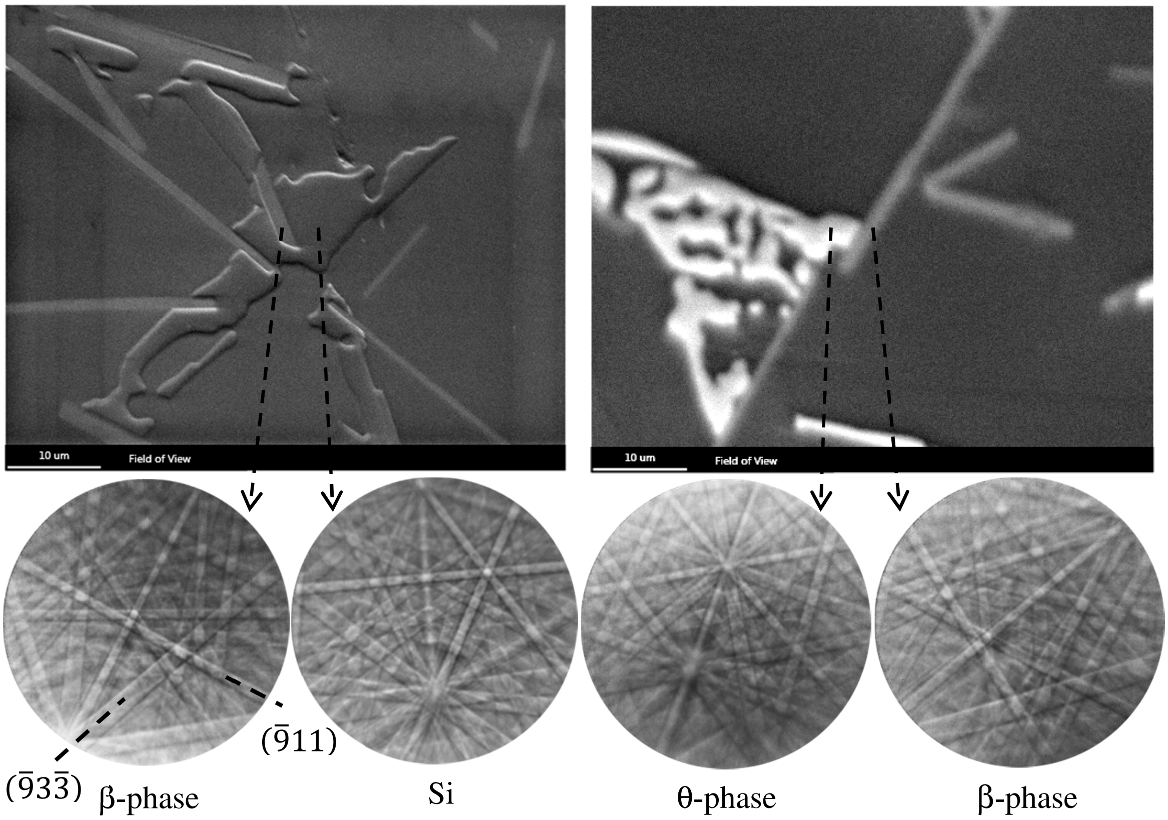

Fig. S1. Typical SEM micrograph and corresponding Kikuchi patterns of β-phase and Si precipitate (left); β-phase and θ-phase (right) in as-cast 6Cu alloy sample. Either $\{933\}$ and/ or $\{\bar{9}11\}$ plane of β-phase exhibit parallelism to different planes of Si precipitates (from different locations) but no such correlation could be established for the β–θ pair (unindexed Kikuchi patterns are presented to reveal the quality of the patterns).

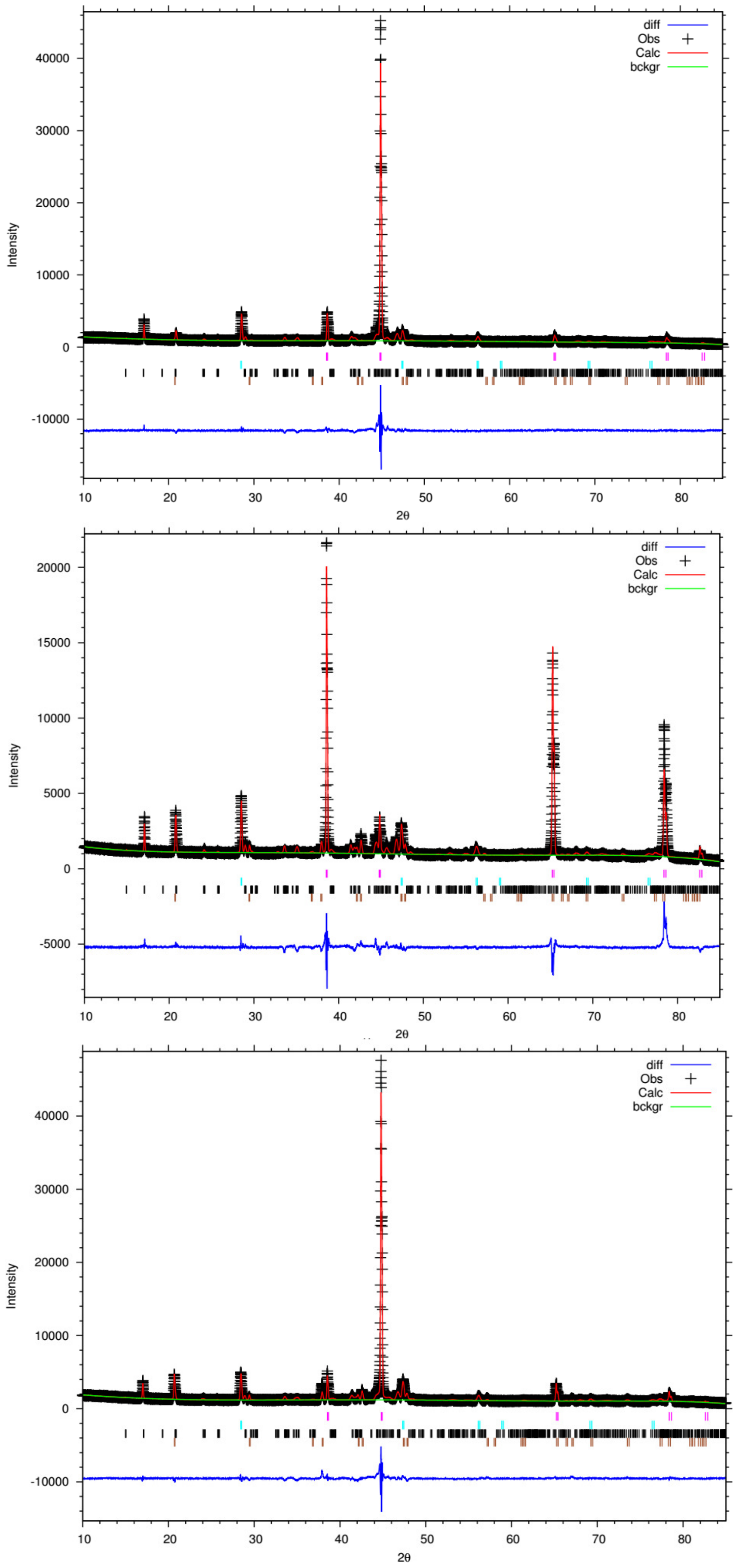

Fig. S2. Experimental and fitted XRD traces for as-cast alloys (from top, 2Cu, 4Cu and 6Cu respectively).

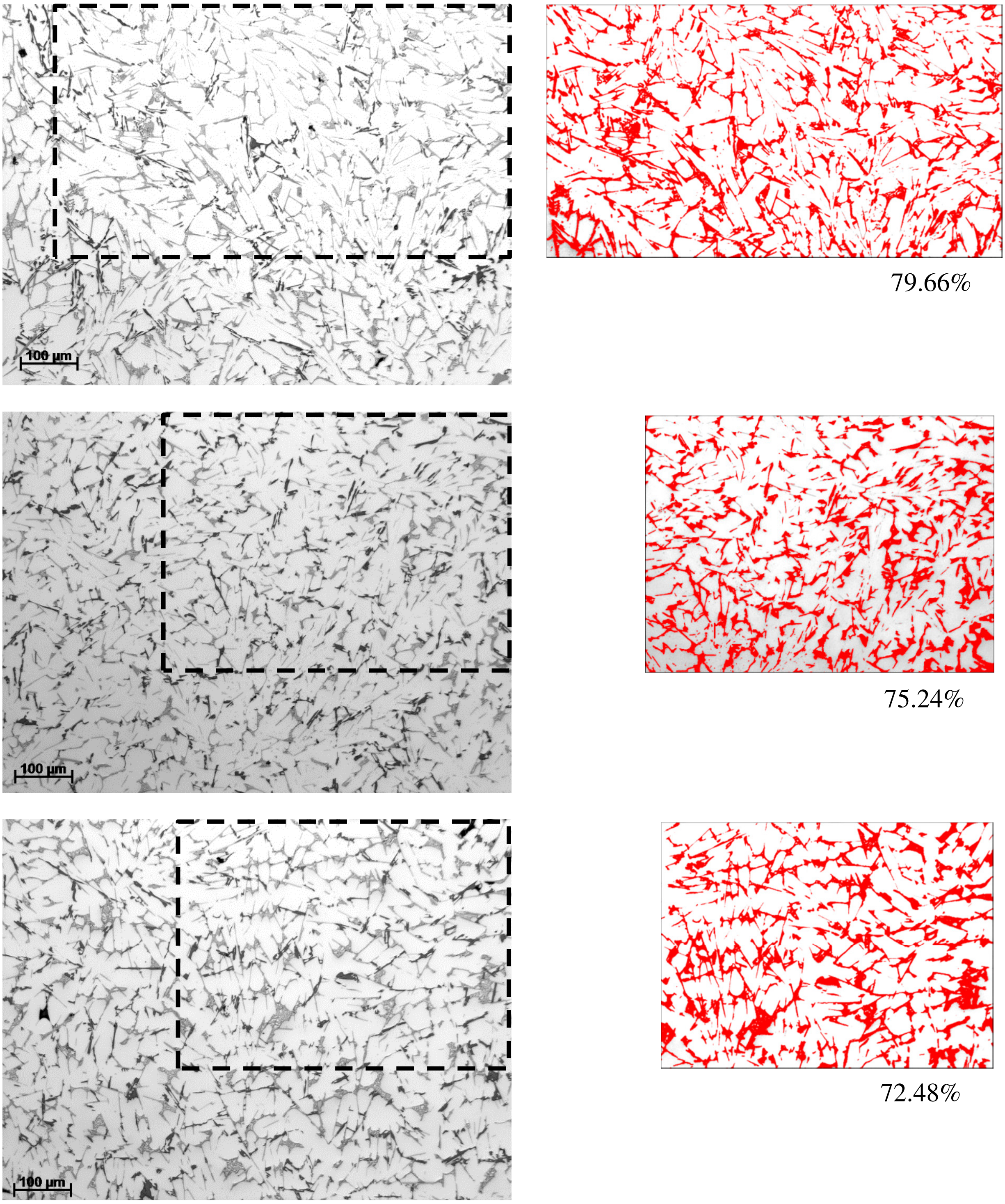

Fig. S3. Optical micrographs of as-cast samples (2Cu, 4Cu and 6Cu alloy from top to bottom, respectively) with selected area for image analysis (left) and corresponding threshold image (right) with calculated area fraction of matrix.

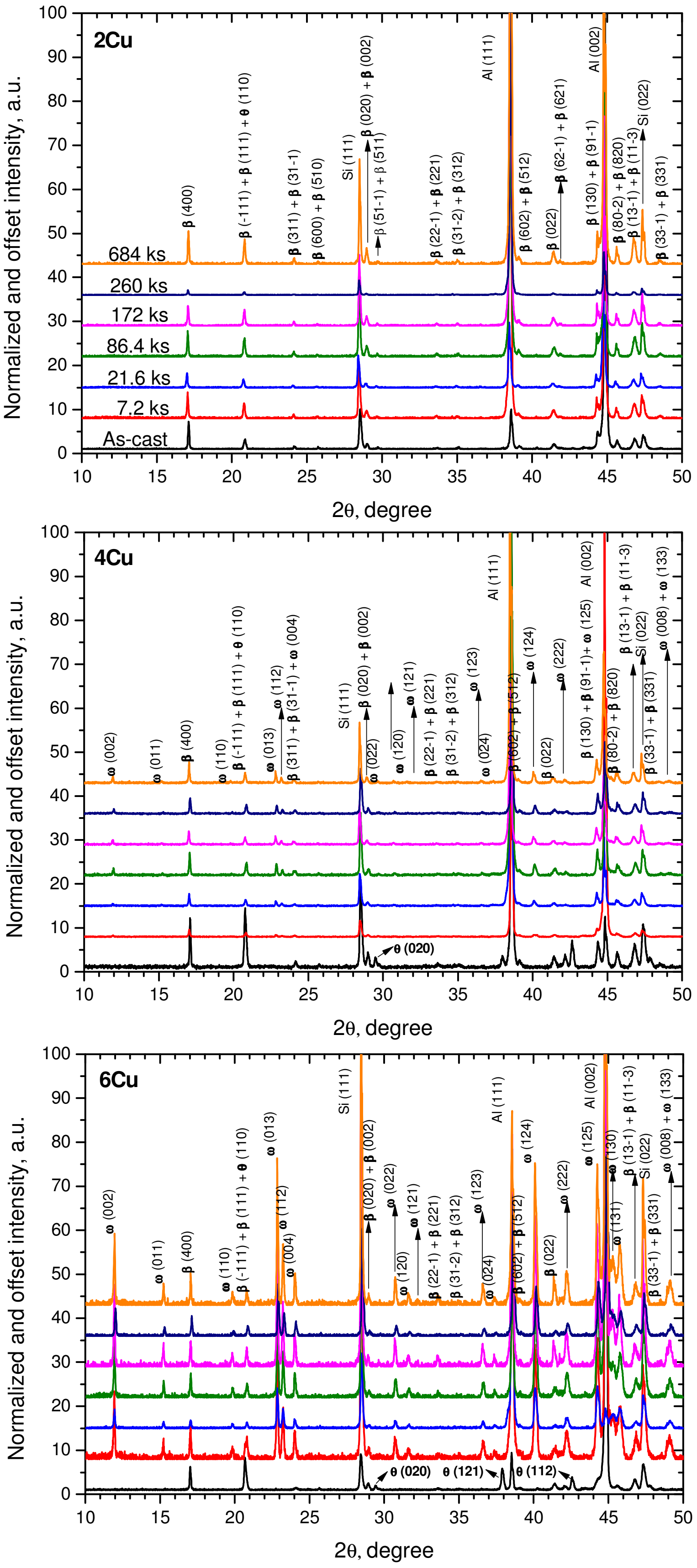

Fig. S4. Indexed XRD traces of solutionized (at 793 K) specimens of 2Cu, 4Cu and 6Cu alloys for different solutionizing time and compared with as-cast sample (black).

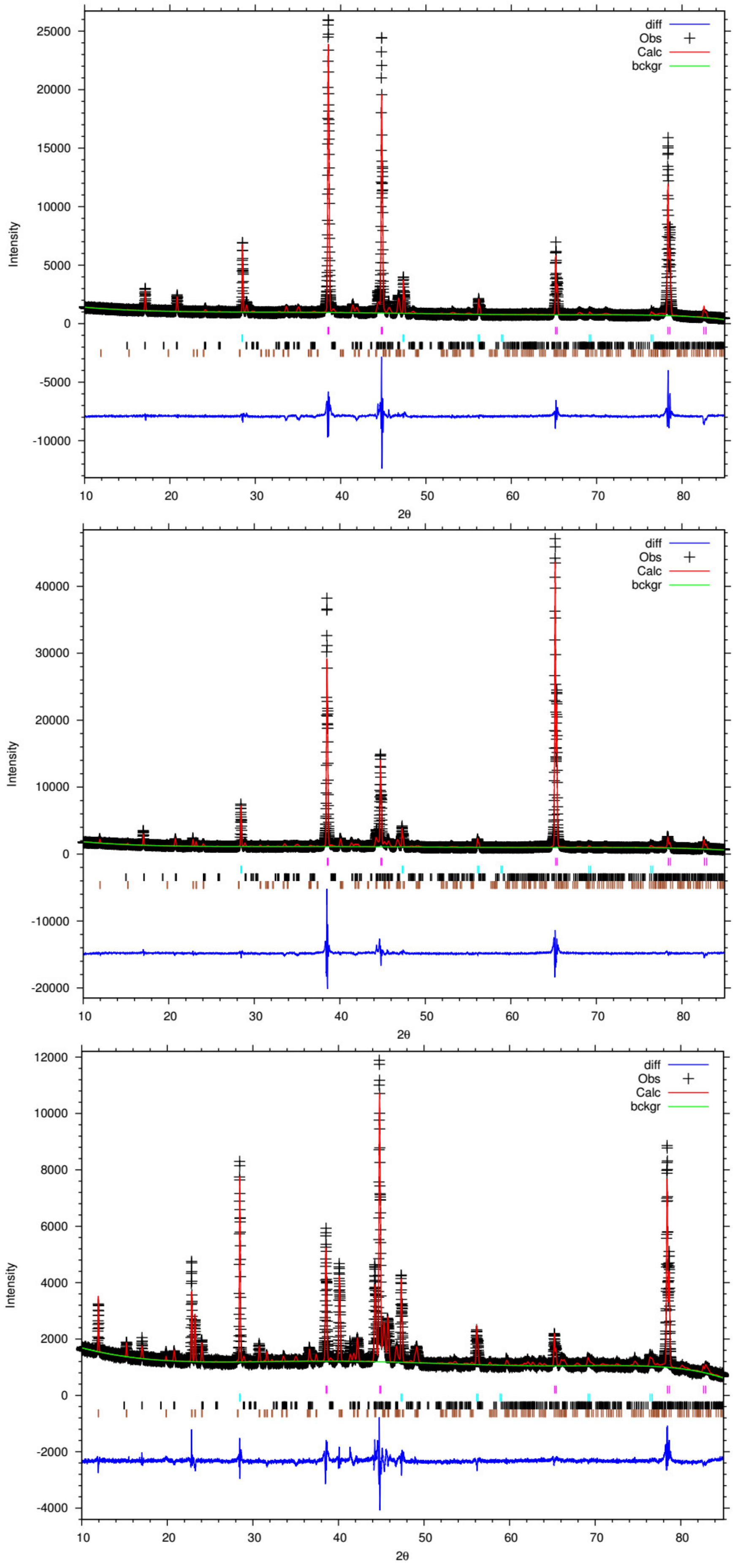

Fig. S5. Experimental and fitted XRD traces for alloys solutionized at 793 K for 684 ks (from top, 2Cu, 4Cu and 6Cu respectively)

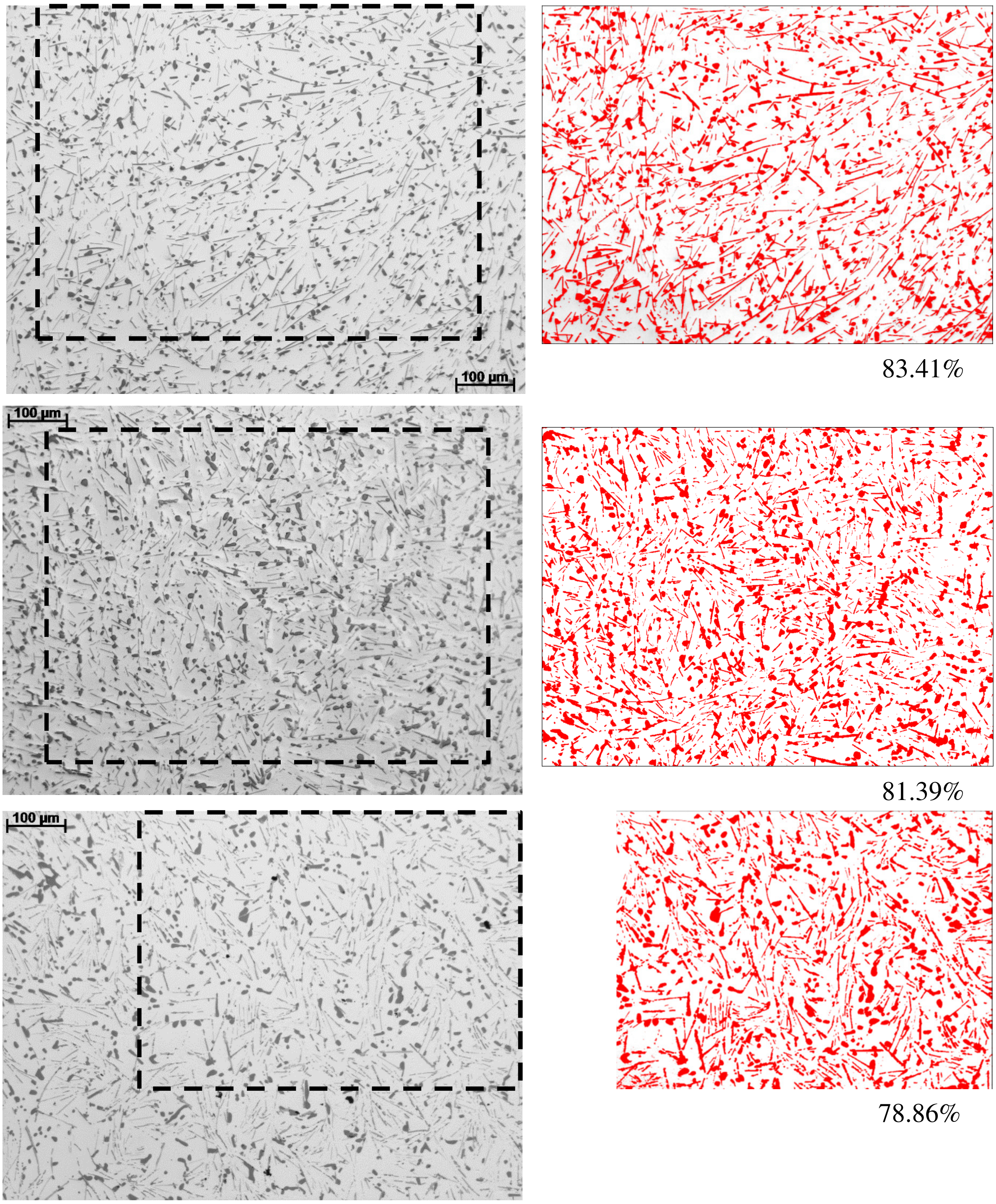

Fig. S6. Optical micrographs of solutionized samples at 793 K for (2Cu, 4Cu and 6Cu alloy from top to bottom, respectively) with selected area for image analysis (left) and corresponding threshold image (right) with calculated area fraction of matrix.

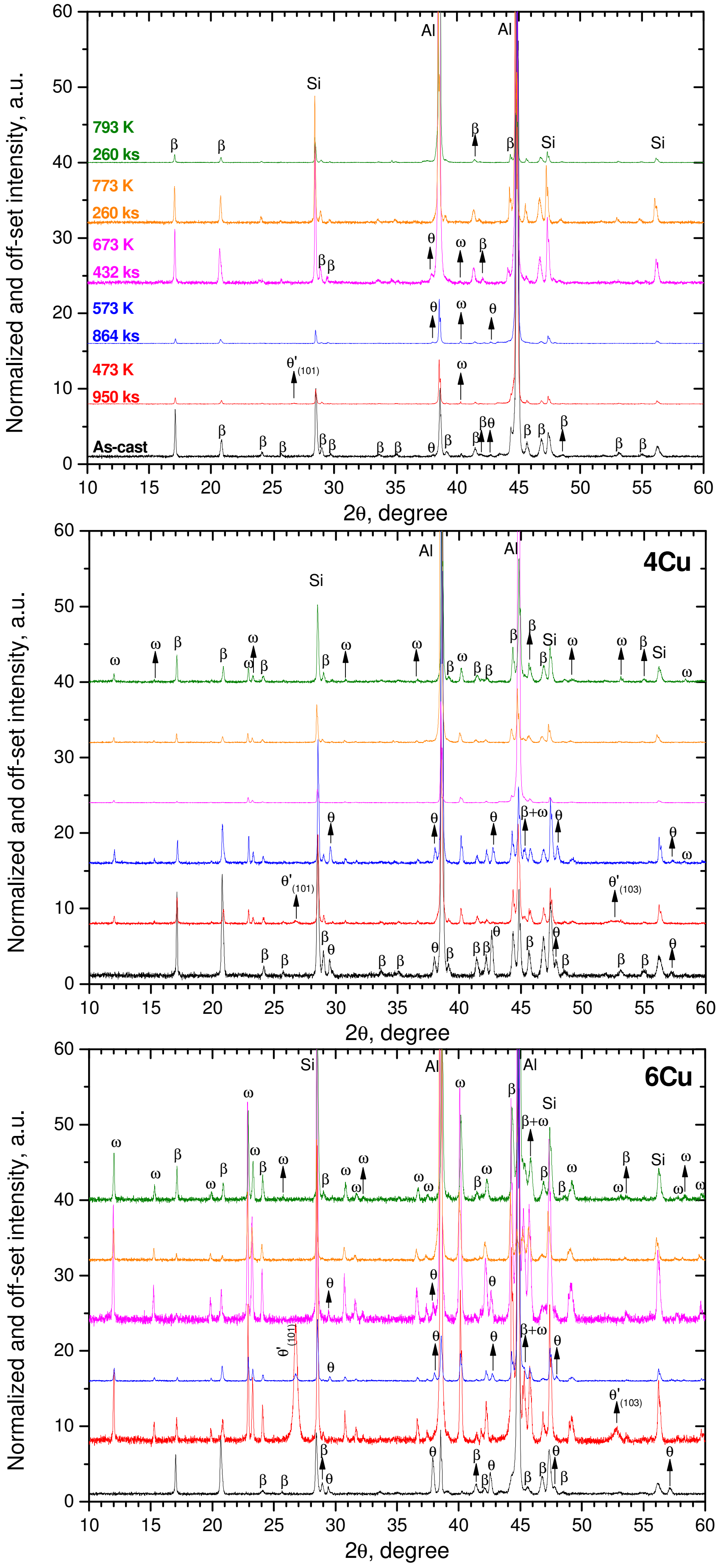

Fig. S7. XRD trace of 2Cu, 4Cu and 6Cu (from top to bottom) samples under different homogenisation temperature.

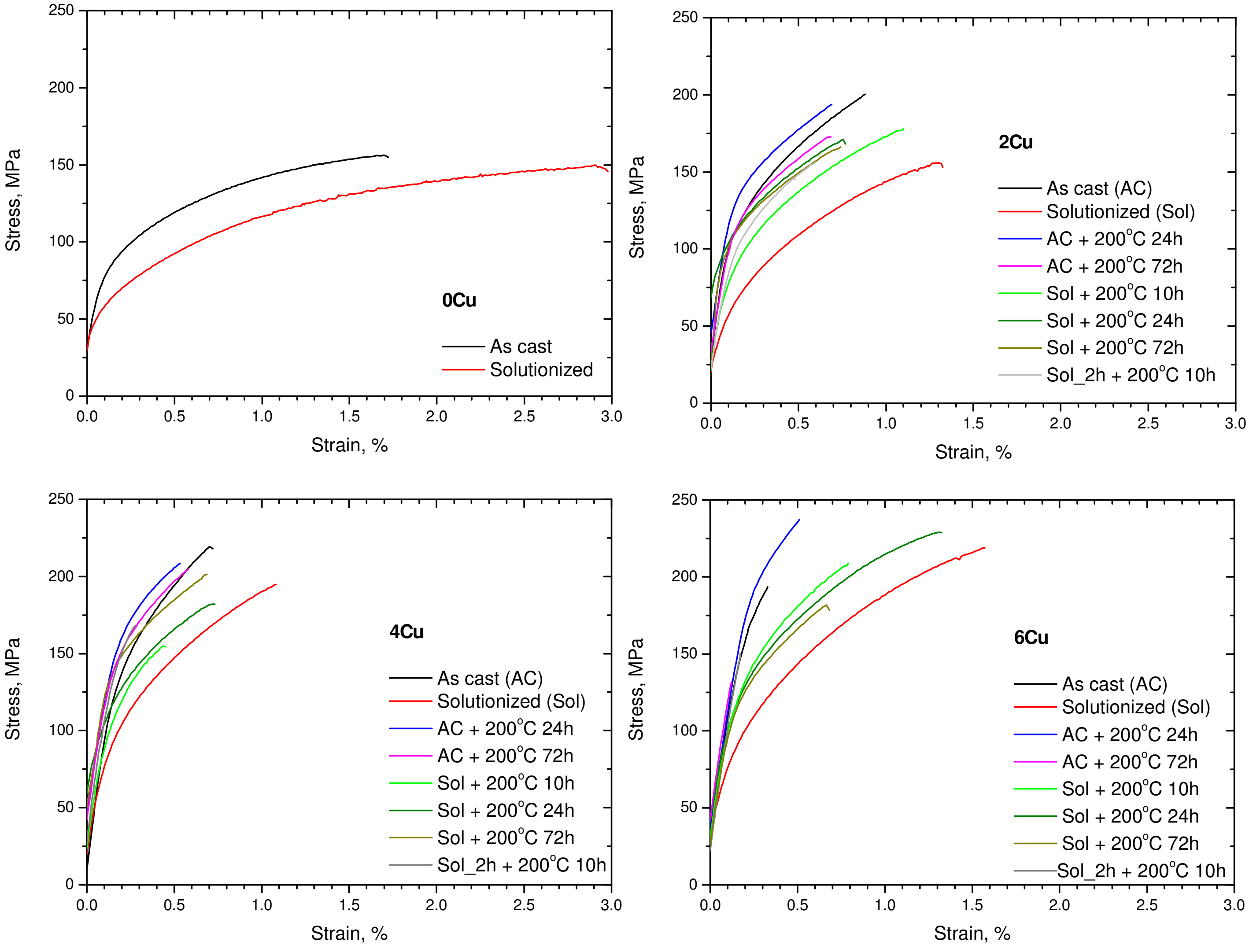

Fig. S8. Stress-strain curves obtained from the 12.5mm gauge length tensile test (only one amongst two tests is shown here).

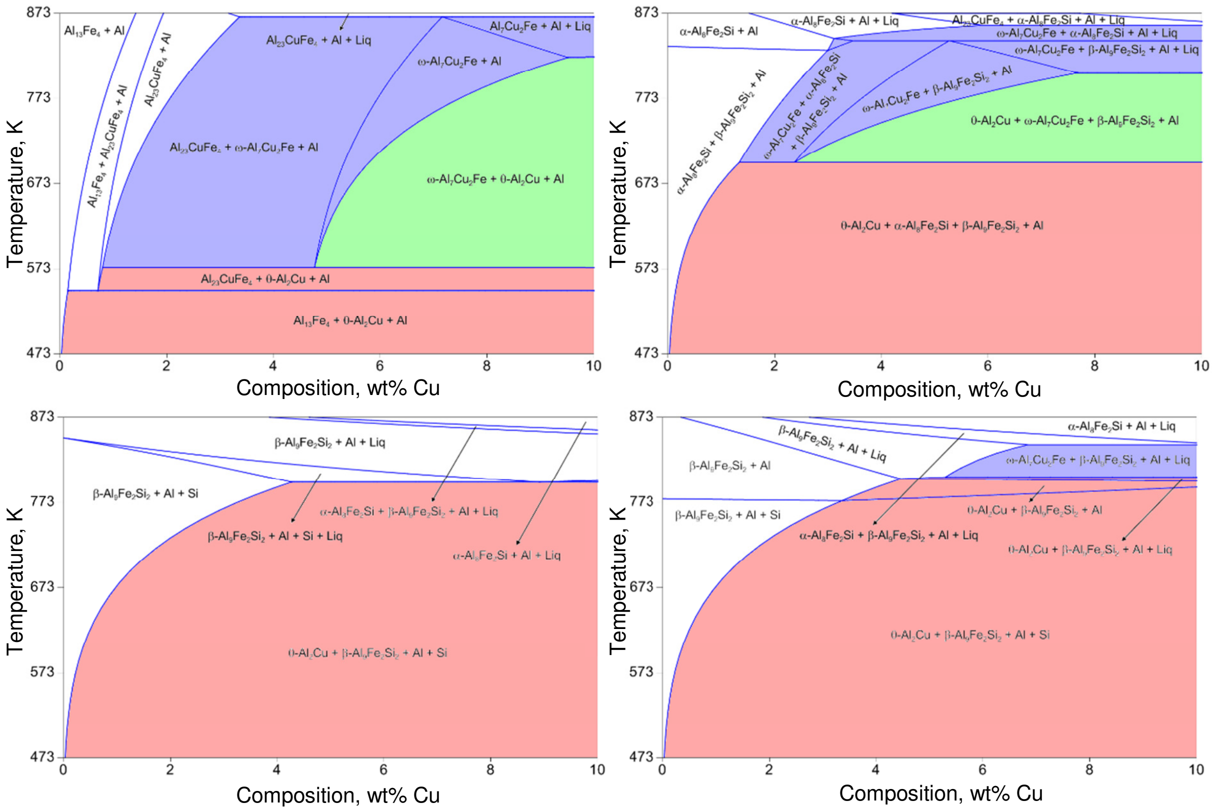

Fig. S9. Computed isopleths at 2wt% Fe for different Si content; namely, (clockwise from the top left) 0wt% Si, 1wt% Si, 2wt% Si and 3wt% Si. Blue and red coloured field denotes presence of ω-Al_7Cu_2Fe and θ-Al_2Cu phase, respectively; whereas green shade indicates co-existence of both. Beyond 3wt% Si, area containing θ-Al_2Cu phase does not change appreciably and the ω-phase field completely disappears.

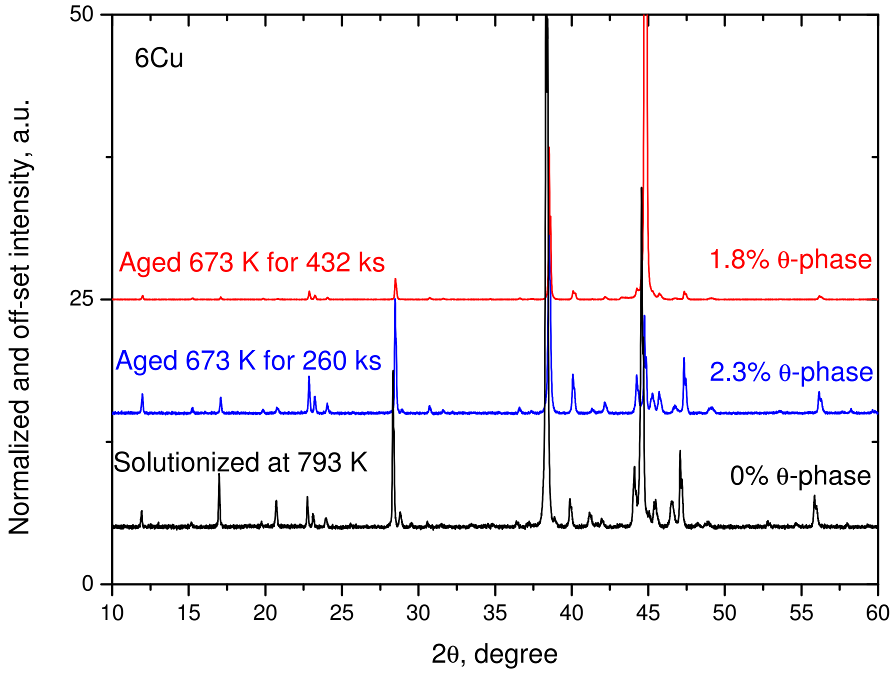

Fig. S10. XRD traces of 6Cu sample aged at 673 K for 260 ks and 432 ks; compared with solutionized condition; Rietveld analysis reveals ω-phase fraction increases monotonously with ageing time from 6.58% (solutionized) to 7.1% (aged for 260 ks) and further to 9.5% (aged for 432 ks). But θ-phase fraction first increases and then decreases with ageing time, indicating its metastable nature at 673 K.
